# Obstetricians and the 2009–2010 H1N1 Vaccination Effort: Implications for Future Pandemics

**DOI:** 10.1007/s10995-012-1104-x

**Published:** 2012-08-22

**Authors:** Sarah J. Clark, Anne E. Cowan, Pascale M. Wortley

**Affiliations:** 1 Child Health Evaluation and Research (CHEAR) Unit, University of Michigan, 300 N Ingalls, Rm 6E06, Ann Arbor, MI 48109-5456 USA; 2 Immunization Services Division, NCIRD, Centers for Disease Control and Prevention, Atlanta, GA USA

**Keywords:** H1N1 virus, Influenza vaccine, Pandemic, Obstetricians

## Abstract

Our objective was to describe the experiences of obstetricians during the 2009–2010 H1N1 vaccination campaign in order to identify possible improvements for future pandemic situations. We conducted a cross-sectional mail survey of a national random sample of 4,000 obstetricians, fielded in Summer 2010. Survey items included availability, recommendation, and patient acceptance of H1N1 vaccine; prioritization of H1N1 vaccine when supply was limited; problems with H1N1 vaccination; and likelihood of providing vaccine during a future influenza pandemic. Response rate was 66 %. Obstetricians strongly recommended H1N1 vaccine during the second (85 %) and third (86 %) trimesters, and less often during the first trimester (71 %) or the immediate postpartum period (76 %); patient preferences followed a similar pattern. H1N1 vaccine was typically available in outpatient obstetrics clinics (80 %). Overall vaccine supply was a major problem for 30 % of obstetricians, but few rated lack of thimerosal-free vaccine as a major problem (12 %). Over half of obstetricians had no major problems with the H1N1 vaccine campaign. Based on this experience, 74 % would be “very likely” and 12 % “likely” to provide vaccine in the event of a future influenza pandemic. Most obstetricians strongly recommended H1N1 vaccine, had few logistical problems beyond limited vaccine supply, and are willing to vaccinate in a future pandemic. Addressing concerns about first-trimester vaccination, developing guidance for prioritization of vaccine in the event of severe supply constraints, and continued facilitation of the logistical aspects of vaccination should be emphasized in future influenza pandemics.

## Introduction

In the US, pregnant women have been a nationally recommended target group for seasonal influenza vaccine since 1997 [1], and epidemiologic research has shown that they have an elevated risk of influenza-related complications [2]. A novel strain of influenza A virus (H1N1) identified in 2009 [3] was also shown to increase health risks for pregnant women [4] and, thus, they were designated as a one of five target groups to receive H1N1 monovalent vaccine during the 2009–2010 influenza season [5].

Data from prior influenza seasons showed that less than a quarter of pregnant women typically received seasonal influenza vaccination [4]. Therefore, broad participation of obstetricians in recommending and offering H1N1 vaccine to pregnant women was viewed as a key to successful vaccination of this high-risk group. While seasonal influenza vaccine for pregnant women is typically purchased in the private sector by outpatient clinics and birthing hospitals, H1N1 vaccine was handled differently due to the pandemic situation. All H1N1 vaccine was supplied free of charge by the federal government; state and local public health officials had the responsibility for coordinating provider enrollment and vaccine distribution. Obstetrician participation in H1N1 vaccination thus hinged on many factors, including outreach from public health officials to ensure that obstetricians were aware of the program and the processes for participation, as well as the willingness of obstetricians to administer the vaccine to pregnant women in the office setting.

We undertook this study to describe the experiences of obstetricians during the 2009–2010 H1N1 vaccination campaign, to assess the extent of their participation and to identify key factors that may inform future pandemic influenza vaccination efforts targeting pregnant women.

## Materials and Methods

### Study Design

The study design was a cross-sectional mail survey of a national random sample of 4,000 obstetricians.

### Survey Administration

We drew the sample from the American Medical Association (AMA) Physician Masterfile; the Masterfile is the most comprehensive database of licensed physicians in the United States and includes both AMA members and non-members. We included physicians with a self-reported primary specialty of obstetrics or obstetrics/gynecology in office-based, direct patient care, and excluded those >70 years, currently in residency training, or employed at federally owned medical facilities (e.g. Veterans Affairs). Masterfile data included mailing address, age, and gender. The initial survey mailing was sent in June 2010 and included a $5 cash incentive. Two additional mailings to non-respondents occurred at approximately 4-week intervals. The institutional review board of the University of Michigan Medical School approved this study.

### Survey Items

The 4-page survey instrument included fixed-choice questions regarding:

- Typical recommendation regarding H1N1 vaccination for obstetric patients in the first, second, and third trimesters, as well as the immediate post-partum period;
- Patient preference for stage of pregnancy in which to receive H1N1 vaccine, relative to their current stage;
- Own and perceived patient level of concern with safety of H1N1 vaccine relative to that of seasonal influenza vaccine;
- Where H1N1 vaccine was available for obstetric patients (e.g. main outpatient clinic);
- Among H1N1 vaccinators, the extent to which eight specific issues related to H1N1 implementation (e.g. lack of thimerosal-free vaccine) were problematic;
- Whether prioritized H1N1 vaccine among obstetric patients when supply was limited and, if so, to which subgroups, and source of information for prioritization decisions;
- Whether practice offered seasonal influenza vaccine in 2009–2010 and whether practice plans to offer influenza vaccine for the 2010–2011 influenza season;
- Likelihood of practice providing vaccine in the event of a future influenza pandemic; and
- Practice characteristics.

### Statistical Analyses

After generating descriptive statistics, we used χ^2^ tests to assess bivariate associations between key outcome variables (availability of vaccine, strength of recommendation, likelihood of providing vaccine during future pandemics) and predictor variables (problems with providing H1N1 vaccine, personal and practice demographics). A two-tailed α-level of 0.05 was the threshold for statistical significance. All analyses were conducted using SAS^®^ version 9.2 (SAS Institute, Cary, NC).

## Results

Of the 4,000 physicians in the mailing sample, 214 were excluded because mailing addresses were incorrect. Returned surveys from 2,486 respondents yielded an overall response rate of 66 %. After excluding 479 respondents who do not provide obstetric care and three surveys received after data analyses had been conducted, our final analytic sample contained 2,004 obstetricians.

### Respondent Characteristics

For practice setting, 67 % of survey respondents were in private independent practice, with another 21 % in practices affiliated with hospitals, health systems or universities, and 12 % in other settings (e.g. public clinics). In terms of practice size, 28 % were in a practice with 1–2 physicians, 32 % with 3–5 physicians, 24 % with 6–10 physicians, and 16 % with more than 10 physicians.

Many respondents reported that seasonal influenza vaccine was available in their practice during the 2009–2010 season (77 %) and would be available for 2010–2011 (78 %).

Gender was evenly split (51 % female, 49 % male). The age distribution was 18 % of respondents <40 years, 51 % between 40 and 55 years, and 31 % >55 years (mean: 49 years). Non-respondents were more likely to be male (*p* = 0.006) and aged 40–55 years (vs. other two age groups, *p* < 0.001).

### Obstetrician Recommendations and Patient Preferences for H1N1 Vaccine

As presented in Table 1, recommendation of H1N1 vaccine was strongest for obstetric patients in the second and third trimesters, and weakest in the first trimester. Younger obstetricians (<40 years) were more likely than those 40–55 years and >55 years (76 vs. 73 vs. 64 %, respectively, *p* < 0.001) and female more likely than male obstetricians (75 vs. 68 %, *p* = 0.003) to recommend H1N1 vaccine in the first trimester.

**Table 1:** Obstetricians’ typical recommendation for H1N1 vaccination (n = 2,004)

For obstetric patients in the…	Strongly recommended vaccination (%)	Recommended vaccination (%)	Neutral (%)	Recommended against vaccination (%)
First trimester	71	18	6	5
Second trimester	86	12	2	<0.5
Third trimester	85	12	2	<1
Immediate post-partum period	76	17	6	<0.5

Obstetricians’ perceptions about patient H1N1 vaccine preferences are presented in Table 2. Half of obstetricians reported that patients in their first trimester preferred to delay H1N1 vaccination until later in pregnancy. In contrast, obstetricians reported strong patient acceptance during the second and third trimesters, as well as during the immediate postpartum period.

**Table 2:** Obstetricians’ perceptions of patients’ preferences for H1N1 vaccination (n = 2,004)

Of my obstetric patients in the…	Most preferred to…			
	Be vaccinated at that stage (%)	Wait until later in pregnancy (%)	Wait until postpartum period (%)	Not be vaccinated at all (%)
First trimester	50	46	<0.5	4
Second trimester	92	6	<0.5	2
Third trimester	92	–	5	3
Immediate post-partum period	88	–	–	12

While only 9 % of obstetricians reported that their own concerns about vaccine safety were greater for H1N1 vaccine than for seasonal influenza vaccine, the majority (56 %) perceived greater vaccine safety concerns among their patients for H1N1 vaccine relative to seasonal influenza vaccine.

### Participation in H1N1 Vaccination Efforts

Ninety percent of obstetricians reported that H1N1 vaccine was available in either their main outpatient obstetrics clinic (80 %) or their birthing hospital (31 %). Only 10 % of obstetricians indicated that H1N1 vaccine was available only at the public health department or other outside location. Availability of vaccine was associated with strength of H1N1 vaccine recommendation: obstetricians who had H1N1 vaccine available at their main outpatient clinic were more likely than those who did not have vaccine at their outpatient practice to strongly recommend vaccination in the first (75 vs. 55 %, *p* < 0.001), second (89 vs. 73 %, *p* < 0.001), and third (88 vs. 72 %, *p* < 0.001) trimesters, and in the immediate postpartum period (80 vs. 61 %, *p* < 0.001).

As shown in Table 3, inconsistent vaccine supply was a significant issue during the H1N1 vaccine campaign, with 30 % of obstetricians rating this as a major problem. Lack of thimerosal-free H1N1 vaccine was viewed as a major problem by only 12 % of obstetricians, while 47 % said it was a minor problem. Almost half of respondents felt low patient acceptance of H1N1 vaccine was a minor problem. Across the issues listed in Table 3, over half of respondents (54 %) reported no major problems with H1N1 vaccination. Obstetricians in private practices were more likely to report three or more major problems compared to their counterparts in hospital- or health system-affiliated settings (6 vs. 3 %, *p* = 0.011).

**Table 3:** Obstetricians’ rating of problems with implementing H1N1 vaccination (n = 2,004)

	Not a problem	Minor problem	Major problem
Inadequate/inconsistent vaccine supply	29	41	30
Lack of thimerosal-free vaccine	41	47	12
Low patient acceptance/demand for vaccine	43	48	9
Staff unfamiliar with H1N1 vaccine products	78	21	1
Storage/handling of vaccine	84	14	2
Reimbursement/billing for vaccine administration	67	26	7
H1N1 reporting requirements	60	33	7
Return/disposal of unused vaccine	70	26	4

Over half (55 %) of obstetricians reported that they prioritized which obstetric patients were offered H1N1 vaccine when supplies were limited. Groups given priority were pregnant women with underlying high-risk conditions (94 % of those who prioritized) and pregnant healthcare workers (76 %). For the 45 % of obstetricians who did not prioritize H1N1 vaccine, the majority (72 %) indicated that they consistently had sufficient vaccine and did not need to prioritize. However, 11 % reported they had no mechanism for determining priorities, and 15 % that it seemed unfair or arbitrary to prioritize.

### Participation in Future Pandemic Influenza Vaccination Efforts

Most obstetricians reported that based on their 2009–2010 H1N1 vaccine experience, they would be *very likely* (74 %) or *likely* (12 %) to provide vaccine in the event of a future influenza pandemic. Another 6 % were *neutral*, 3 % were *unlikely*, and 5 % were *very unlikely*.

Participation in a future pandemic was associated with practice setting, size of practice, availability of seasonal influenza, and H1N1 vaccine availability. Obstetricians in hospital- or health system-affiliated practices were more likely than those in independent private practice to be *very likely* to participate in a future pandemic (82 vs. 67 %, *p* < 0.001), as were obstetricians who offered vs did not offer seasonal influenza vaccine during 2009–2010 (70 vs. 26 %, *p* < 0.001). In addition, participation in a future pandemic event was more likely for those who did vs did not have the 2009 H1N1 vaccine available at their main outpatient practice (78 vs. 30 % *p* < 0.001).

Obstetricians’ willingness to provide vaccine during a future influenza pandemic event declined with an increasing number of major problems experienced with H1N1 vaccination: 85 % of those reporting no major problems indicated they would be *very likely* to vaccinate in a future influenza pandemic, compared to only 44 % of those who reported three or more major problems (*p* < 0.001).

## Discussion

At the outset of the 2009–2010 H1N1 vaccination campaign, obstetricians represented a potentially important point of access to H1N1 vaccine for pregnant women. However, the extent to which obstetricians would be involved in the campaign was unclear, as seasonal influenza vaccination rates among pregnant women had languished at <15 % since first recommended in 1997 [1, 2, 6]. This national survey of obstetricians demonstrates that access to H1N1 vaccine among obstetricians was widespread. These findings, confirmed by other research [7], also show that the rate of H1N1 vaccine availability in the practice setting was higher than that seen previously for seasonal influenza vaccine [8–10], and was slightly higher than the reported availability of seasonal influenza in their practices for the 2009–2010 and 2010–2011 seasons. Obstetrician involvement may have been influenced by early reports of influenza-related mortality in pregnant women [11], as well as coordinated efforts by public health officials and professional societies, such as the American College of Obstetricians and Gynecologists (the College), to communicate to providers the importance of participating as H1N1 vaccinators [12]. In a pandemic situation, having vaccine in the obstetric setting is an efficient way to target pregnant women, and the majority of pregnant women who received H1N1 vaccine were vaccinated in the obstetric setting [13, 14].

Consistent with our finding that the majority of obstetricians strongly recommended H1N1 vaccine to their patients, vaccine coverage data indicate that almost half of pregnant women were vaccinated with the H1N1 vaccine [13, 15, 16]. Provider recommendation was a key factor associated with receipt of H1N1 vaccine by pregnant women [13, 15, 17, 18]. However, the lower proportion of obstetricians who strongly recommended first-trimester vaccination, as well as the perceived patient reluctance toward first-trimester vaccination, observed in this survey is consistent with lower first-trimester recommendations [7] and vaccination rates shown previously [9, 19]. This may reflect the fact that first-trimester vaccination was not recommended until the 2004–2005 influenza season [20]. It also may reflect the general lack of data about the safety of vaccination at this stage of pregnancy. As a broader base of both scientific and empirical data is established, obstetricians and patients may become more comfortable with first-trimester vaccination. Helping obstetricians translate relevant information for patients, such as recent data demonstrating the benefits of maternal influenza vaccination for infants’ health [21–23], is an important point of emphasis for future pandemic situations.

Vaccine supply was the main barrier for obstetricians, even though pregnant women were one of the five sub-populations specifically targeted for vaccination [5]. When vaccine supply was limited in the early stages of the vaccination campaign, state and local health departments—who had responsibility for allocation of vaccine—varied widely in the populations and provider types prioritized in their allocation. Moreover, the first doses to become available were live attenuated intranasal vaccine (LAIV) [24], which cannot be given to pregnant women; it is plausible that obstetricians and pregnant women were frustrated that H1N1 vaccine doses were available, but not available to pregnant women. In subsequent weeks, as a limited supply of inactivated doses became available, obstetricians who were given vaccine faced decisions on how to allocate the limited amount within their patient population. Results from this survey indicate that when obstetricians prioritized which pregnant patients would receive H1N1 vaccine, they generally focused on those who were in more than one of the five vaccine target groups in the national recommendations (e.g. pregnant patients who were also healthcare workers) [5]. For future pandemic situations, clear guidance (for example, indicating that providers with insufficient supply should prioritize based on number of risk factors or severity of high-risk condition) would be helpful. The finding that relatively few obstetricians reported having a major problem with lack of thimerosal-free vaccine indicates that concerns about thimerosal may be outweighed by the desire for timely vaccination during a public health emergency.

Practical aspects of vaccination in the practice setting, including reimbursement and billing, were rated as non-problems by the majority of obstetricians. The relative ease of these logistical issues is important to future pandemic situations: while the vast majority of obstetricians responding to this survey indicated that they would be willing to provide vaccine in a future influenza pandemic, those who reported major problems with H1N1 vaccination (beyond vaccine supply issues) were substantially less willing to vaccinate in a future pandemic. Thus, there appears to be both a present and future benefit to facilitating obstetricians’ involvement in influenza vaccination. More broadly, it seems likely that any type of public health emergency in which pregnant women would be considered a priority patient population would benefit from the participation of obstetricians, both in preparedness efforts (e.g. planning, training) and implementation of the actual emergency response.

Some limitations are noted for this study. These results represent self-reported attitudes, behaviors and practices, and the study was not designed to independently verify the accuracy of self-report. Also, as is inherent with mail surveys, there is the potential for response bias. Though our analyses demonstrated minimal differences in demographic characteristics between respondents and non-respondents, obstetricians who did not provide H1N1 vaccine may have been less likely to respond. While we cannot ascertain the magnitude of this bias, the response rate for this study compares favorably with other recent, national surveys of obstetricians (42–66 %) [7, 25–29]. In addition, the implications of our findings for improving the experiences of obstetricians participating in future pandemic vaccination efforts are unlikely to be affected by lower response from those who did not provide H1N1 vaccine.

In conclusion, given the generally positive experiences of obstetricians in the 2009–2010 H1N1 vaccination campaign, and the H1N1 vaccination rates for pregnant women, public health officials should continue to include obstetricians in their planning efforts around pandemic influenza. Addressing concerns about first-trimester vaccination, developing guidelines for sub prioritization of vaccine in the event of severe supply constraints, and continued facilitation of the logistical aspects of vaccination should be emphasized in future influenza pandemics.
